# P258: Incidence and risk factors of bacteremia associated with care intensive care environment: study in Chu Sahloul (Sousse Tunisia)

**DOI:** 10.1186/2047-2994-2-S1-P258

**Published:** 2013-06-20

**Authors:** D Chebil, M Ben Rejeb, H Latiri, N Jaidane, S Khefacha, M Miladi, K Ben Alaya, L Dhidah

**Affiliations:** 1 Microbiology Laboratory, Hospital of Sahloul, Sousse, Tunisia

## Introduction

In the context of the fight against healthcare associated infections (IAS) in intensive care, the CLIN in collaboration with hospital hygiene service, have established a system of epidemiological surveillance to investigate the risk factors of IAS including bacteremia (BAS).

## Methods

This is a longitudinal study conducted in the University Hospital Intensive Care services Sahloul during 2010 - 2011. We included all patients hospitalized for more than 48 hours. This study is based on the French national protocol GRAPE-REA 2004. Analysis of data was performed by the software SPSS 19.0.

## Results

We included 301 patients. Among them, 21 had BAS with an estimated incidence of 7%. The univariate analysis identified as risk factors: age, SAPS II, and duration of intubation, tracheotomy, urinary catheterization and the duration, central venous catheterization and its duration.

In addition, multivariate analysis highlighted intubation and tracheostomy as independent risk factors.

## Conclusion

Invasive devices play a major role in the development of BAS. They urge us to review the practices in intensive care units related to indications of these devices.

## Competing interests

None declared

